# GPNMB Ameliorates Neuroinflammation Via the Modulation of AMPK/NFκB Signaling Pathway After SAH in Mice

**DOI:** 10.1007/s11481-023-10087-6

**Published:** 2023-11-03

**Authors:** Tao Li, Yuansheng Zhang, Qixiong Lu, Li Lei, Jingshu Du, Xiaoyang Lu

**Affiliations:** 1 Department of Neurosurgery, The First People’s Hospital of Yunnan Province, The Affiliated Hospital of Kunming University of Science and Technology, Kunming, Yunnan China; 2 Department of Neurosurgery, The Affiliated Hospital of Kunming University of Science and Technology, The First People’s Hospital of Yunnan Province, Kunming, Yunnan China; 3 Department of Traditional Chinese Medicine, The First People’s Hospital of Yunnan Province, The Affiliated Hospital of Kunming University of Science and Technology, Kunming, Yunnan China

**Keywords:** Glycoprotein non-metastatic melanoma protein B, Subarachnoid hemorrhage, Early brain injury, Neuroinflammation, Neurofunction

## Abstract

Glycoprotein non-metastatic melanoma protein B (GPNMB) got its name from the first discovery in a cell line of non-metastatic melanoma. Later studies found that GPNMB is widely expressed in various tissues and cells of the human body, most abundant in neural tissue, epithelial tissue, bone tissue, and monocyte-macrophage system. GPNMB has been shown to have anti-inflammatory effects in a variety of neurological diseases, however, it has not been reported in subarachnoid hemorrhage (SAH). Male CD-1 mice were used and intra-arterial puncture method was applied to establish the SAH model. Exogenous recombinant GPNMB (rGPNMB) was injected intracerebroventricularly 1 h after SAH. SAH grading, brain edema and blood–brain barrier (BBB) integrity were quantified, and neurobehavioral tests were performed to evaluate the effect of GPNMB on the outcome. Dorsomorphin, the selective inhibitor on AMPK was introduced to study the downstream signaling through which the GPNMB works. Furthermore, western blot, immunofluorescence staining and ELISA were utilized to confirm the signaling. After SAH, GPNMB expression increased significantly as a result of the inflammatory response. GPNMB was expressed extensively in mouse microglia, astrocytes and neurons. The administration of rGPNMB could alleviate brain edema, restore BBB integrity and improve the neurological outcome of mice with SAH. GPNMB treatment significantly magnified the expression of p-AMPK while p-NFκB, IL-1β, IL-6 and TNF-α were suppressed; in the meantime, the combined administration of GPNMB and AMPK inhibitor could decrease the intensity of p-AMPK and reverse the quantity of p-NFκB and the above inflammatory cytokines. GPNMB has the potential of ameliorating the brain edema and neuroinflammation, protecting the BBB and improving the neurological outcome, possibly via the AMPK/NFκB signaling pathway.

**Graphical Abstract:**

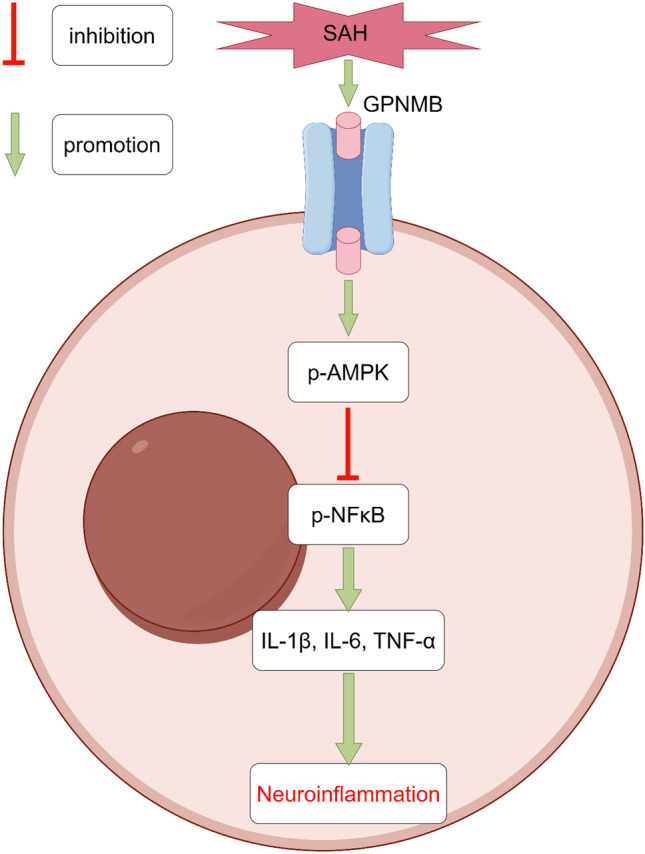

**Supplementary Information:**

The online version contains supplementary material available at 10.1007/s11481-023-10087-6.

## Introduction

Subarachnoid hemorrhage (SAH) is a kind of hemorrhagic stroke and a common acute and severe syndrome in neurosurgery with high mortality and disability rate (Xu et al. 2021a, b). The mechanism of brain injury induced by SAH is not clear. In the past, it was thought that cerebral vasospasm after blood contact is the basic principle, and a large number of studies have also focused on it (Fan et al. 2021; Neifert et al. 2021). However, the clinical trials of preventing and treating cerebral vasospasm after SAH were in vain (Shen et al. 2013). In order to promote the research on the mechanism of SAH injury, the latest viewpoint integrates the brain injury occurring within 72 h after SAH into "early brain injury (EBI)", which not only includes cerebral vasospasm, but also covers the pathological processes such as increased intracranial pressure, oxidative stress injury, excessive activation of inflammation and neuronal apoptosis (Li et al. 2019; Xu et al. 2021a, b). This view holds that EBI is the cause of poor prognosis of SAH, and reducing EBI is the key to treating SAH.

Glycoprotein non-metastatic melanoma protein B (GPNMB) was first identified in a cell line of non-metastatic melanoma and named after this (Diaz-Ortiz et al. 2022). Later studies found that GPNMB is widely expressed in various kinds of tissues and cells of the human body, predominantly abundant in neural tissue, epithelial tissue, bone tissue and the monocyte-macrophage system (Saade et al. 2021). GPNMB is a type I transmembrane glycoprotein encoded by the *Gpnmb* gene in humans, and the homologous gene products in mouse and rat are called DC-HIL (Chung et al. 2020) and osteoactivin (OA) (Sondag et al. 2016), respectively. The *Gpnmb* gene and its homologous gene in mammals are evolutionarily conserved, and the structure and function of the protein products are similar. Not only are they involved in basic physiological processes such as nervous system inflammation and cell survival (Neal et al. 2018), epithelial tissue angiogenesis (Narasaraju et al. 2015), bone tissue regeneration and reconstruction (Huang et al. 2021), regulation of immune response and cell signal transduction (Zhang et al. 2019a, b), but also in pathological processes including tumor genesis, metastasis and resistance (Liguori et al. 2021). In recent years, some scholars have found that GPNMB-expressing microglia were significantly increased and activated in the brain tissue of patients with Alzheimer's disease (AD), suggesting that GPNMB may have a connection with neuroinflammatory response in AD (Huttenrauch et al. 2018). Murthy MN et al. reported that elevation of GPNMB expression in brain tissue is related with an increased incidence of Parkinson's disease (Murthy et al. 2017). Similar findings were also reported by Oeckl P et al. that the protein level of GPNMB was significantly increased in the cerebrospinal fluid (CSF) of patients with amyotrophic lateral sclerosis (Oeckl et al. 2020). Thus, it is speculated that GPNMB is closely associated with inflammatory responses in neurodegenerative diseases. Research by Neal ML et al. pointed out that GPNMB alleviates astrocyte-associated neuritis by acting on astrocyte surface receptor CD44 (Neal et al. 2018). Noda Y et al. confirmed that GPNMB can alleviate endoplasmic reticulum stress by inducing binding immunoglobulin protein (BiP), thereby regulating inflammation, apoptosis and other processes, and exerts neuroprotective effects (Noda et al. 2017). Research by Ripoll VM et al. further recognized that GPNMB increases synchronously with the inflammatory response, but GPNMB is not the cause of inflammation, instead it is activated by γ-interferon and acts as a negative feedback regulator of inflammation to reduce excessive inflammatory response (Ripoll et al. 2007).

Some scholars studying acute kidney injury found that GPNMB can facilitate transformation of macrophages into M2 (anti-inflammatory) type to balance the inflammatory response by M1 (pro-inflammatory) type, thereby exerting an anti-inflammatory effect (Zhou et al. 2017). Microglia in the brain tissue are derived from the monocyte-macrophage system in mesoderm and play the roles of phagocytosis, chemotaxis, and inflammatory responses, and it is the most important player in the inflammation of the central nervous system; at the same time, after the occurrence of SAH, a large number of mononuclear-macrophages are in direct contact with brain tissue and also involved in the inflammatory process. Logically, GPNMB can also transform microglia and inflammatory cells that exuded to brain tissue into anti-inflammatory type to rebalance hyperactivated inflammation. More and more studies have revealed that AMPK/NFκB dependent signaling pathway is one of classic pathways of anti-inflammatory transformation of monocyte-macrophages (Ji et al. 2018; Xu et al. 2018).

Taken together, we speculate that GPNMB can play a role in the EBI phase after SAH by activating AMPK dependent signaling pathway to attenuate the inflammatory response.

## Methods Committee

### Animals

Two hundred and twenty male ICR mice (32 ± 5 g, 56 ± 5d old, Vital River, Beijing) were used in our study. The mice could forage the fodder and water ad libitum, and were housed in an animal room (20–25 ℃ temperature, 50–60% humidity) with virtual-nature 12 h light and dark cycles. All the management on the mice complies with the Rules of Laboratory Animal Care and Use, Kunming University of Science and Technology, and at the same time observed the National Institutes of Health (NIH) Guide for the Care and Use of Laboratory Animals.

### SAH Model

Intra-arterial puncture method was adopted to establish the SAH model (Li et al. 2019). Briefly, the mouse to be operated on was anesthetized with a mixture (20 ml/kg, *i.p.*) of ketamine (100 mg/kg) and xylazine (10 mg/kg), and then put in a supine position. Normally, after an incision along the middle line of the neck was made, the left carotid artery (CA), the external carotid artery (ECA) and internal carotid artery (ICA) were dissected. The CA and ICA were momentarily clipped and the ECA was permanently ligated, and then a minor incision was incised on the ECA to insert a 5–0 nylon thread. After the removal of the clip, the thread went along the ICA to reach the bifurcation of middle cerebral artery (MCA) and anterior cerebral artery (ACA) at the time when contacting the obvious resistance. The thread was pushed forward approximately 2 mm and the vessel wall could be punctured. Finally, the thread was retreated, the proximal ECA was ligated and the surgical wound was sutured.

### Grade of SAH

The severity of SAH was graded according to the Sugawara method (Li et al. 2019; Sugawara et al. 2008). As previously described, the basal cistern is divided into 6 areas, and each area may be scored 0 (no blood) to 3 (all arteries full of blood clot). The total score ranges from 0 – 18, and thus the severity of SAH is categorized into mild (0–7), moderate (8–12) and severe (13–18). In our study, the successful model establishment should be the severe SAH.

### Intra-Cerebroventricular Injection

As was described, the anesthetized mouse was fixed to the stereotactic frame, head in a prone position. Taking the bregma the origin, the coordinate (anterior–posterior 1.0 mm, right medial–lateral 1.0 mm, dorsal–ventral depth 2.0 mm) was referred to as the injection point. The recombinant human GPNMB (rGPNMB) protein (2550-AC-050, R&D Systems) was reconstituted in phosphate buffer saline (PBS, 0.01 M) to concentration of 1 µg/10 µL, 3.3 µg/10 µL and 10 µg/10 µL, respectively. Intra-cerebroventricular injection was executed 1 h after SAH induction using a Hamilton micro-injection system at a rate of 2 µL/min; the needle stayed still for 5 min in case of the backflow and pulled back slowly. The drill was sealed with bone wax.

### Intravenous Drug Administration

The selective p-AMPK inhibitor, Dorsomophin (BML-275, S7306, water soluble type, Selleck, USA) was dissolved in PBS (10 mg/kg) and injected into the tail vein under the assisted with the tail vein injection device 1 h after SAH induction.

### Neurofunctional Tests

#### Modified neurological severity scores

The modified neurological severity scores (mNSS) is the most common test of neuronal deficits for mice or rats after stroke. The test is a composition of motor, balance and reflex tests. For mice, the score ranges from 0 to 14, in which 14 means the most severe neuronal deficits while 0 is intact neurofunction.

#### Garcia Test

Garcia test constitutes six trials, including spontaneous activity, symmetry of four limbs, forepaw outstretching, ability to climb, proprioception, and response to vibrissae. Each trial can be scored 0–3, where 0 means no function and 3 means intact function. The test score is calculated from 0 to 18.

#### Rotor-Rod Test

The Rotor-Rod test, either named rotarod test, is used to assess sensorimotor coordination and motor learning, and is commonly applied in the neurofunctional test in rodent models with CNS disorders. In our study, fixed rotarod was used to evaluate the mid- and long-term neuronal function. The device was set on the rotating speed at 5 revolutions per minute (RPM) and 10 RPM without acceleration (Li et al. 2020).

#### Morris Water Maze Test

The test was performed as we described (Li et al. 2020). All the mice were assessed at the baseline and 21 days after SAH, and continued for 6 days. On the first test day (Day 1), the platform was placed in the block (quadrant) same as the baseline. From the second test day (Day 2), the platform was removed and placed in an either clockwise or count-clockwise order to perform the test. The platform was removed on the last test day (Day 6), and the mice were free to swim for 1.5 min under the tracking system to calculate the retaining time in the target block and generate the heatmap.

### Brain Water Content

Direct measure was used in our study to calculate the brain water content (BWC) (Li et al. 2019). The euthanized mouse had the brain dissected immediately without perfusion, and the cerebellum and brainstem removed. The remaining cerebrum was weighted to get the wet weight (WW). Then, it was conveyed to the electrothermostatic oven to be dried out to get the dry weight (DW). The brain water could be quantified using the percentage of the subtraction of WW and DW by the WW, which is BWC = [(WW-DW) / DW] × 100%. BWC is the quantified interpretation of brain edema.

### Blood–Brain Barrier Integrity

Non-toxic Evans blue dye (1%, 10 ml/kg) was injected intravenously into the mouse (Li et al. 2019). One hour later, the deep anesthetized mouse had the transcardial perfusion with PBS (0.01 M, pH 7.40, 4 ℃). The acquired brain had the cerebellum and brainstem removed and was homogenized with 3.0 ml trichloroacetic acid (TCAA), and then centrifugated at 12,000 RPM for 30 min. The supernatant was added into the TCAA-ethanol mixture (TCAA:ethanol = 1:3) and then incubated overnight at 4 ℃. The mixture was again centrifugated at 12,000 RPM for 30 min; the resultant supernatant was examined using a spectrofluorophotometer.

### Western Blot

Western blot (WB) was utilized to detect and analyze the proteins as described (Li et al. 2019, 2020). In short, the left cerebral cortex which was the same side of the surgical puncture was homogenized and centrifugated at 4 ℃ (12,000 RPM) for 30 min because this brain region has the severest SAH induced injury and the most typical pathological reactions, and the supernatant was determined using an assay kit to the equivalent concentration as per the manufacturer’s instruction (Bio-Rad Laboratories, Hercules, CA, USA). Then, the supernatant with the same protein concentration was denature in the hot water bathing at 95 ℃ for 5 min to yield the protein samples. Each time, the protein samples with same volume (10 µl) were loaded in the well of the gel to have the electrophoresis, and the gel containing the separated proteins had transfer procedure using a PVDF membrane. After protein transfer, the PVDF membrane was rinsed with Tris-buffered saline and Tween-20 (TBST) and soaked in with non-fat milk (5%) for 1 h. The primary antibodies were diluted as per the manufacturer’s instructions and added to the PVDF membrane to co-incubate overnight (12 h, 4 ℃). The PVDF membrane was then rinsed with TBST and co-incubated with secondary antibodies (2 h, 25 ℃). In completion of incubation, the membrane was rinsed and processed with ECL reagent. The final membrane was exposed on photosensitive films and the subsequent protein bands were analyzed using the ImageJ software (National Institutes of Health, MD, USA). The primary antibodies used in our study were anti-p-AMPK (1:1000, #5759, Cell Signaling Technology, USA), anti-APMK (1:1000, #5831, Cell Signaling Technology, USA), anti-p-NFκB (1:2000, ab16502, ABCAM, USA), anti-IL-1β (1:2000, ab234437, ABCAM, USA), anti-IL-6 (1:2000, ab233706, ABCAM, USA), anti-TNF-α (1:2000, ab6671, ABCAM, USA), and anti-β-actin (1:4000, ab8226, ABCAM, USA). The corresponding secondary antibodies were anti-rabbit (1:5000; ab205718, Abcam, USA) and anti-mouse (1:5000; ab205719, Abcam, USA).

### Enzyme Linked Immunosorbent Assay

Enzyme linked immunosorbent assay (ELISA) was used to further confirm the expression of above inflammation related cytokines before and after SAH. The process of measurement was as per the manufacturer’s protocol and previous studies (Li et al. 2020; Zhang et al. 2019a, b). The left hemisphere cortex tissue suspension and protein samples were acquired similar to the WB method. The protein samples and the standard were mixed with antibody cocktail in pre-coated 96-well microplate, and then incubated at room temperature (25 ℃) for 1 h. After washing with buffer reagent, the development solution was added to each well, and finally the stop solution was added. Using a spectrophotometer to analyze the optical density (OD) at 450 nm for each well in the microplate, the protein concentration could be quantified by comparing the OD of target protein with standard curve. ELISA kits in the study were used: IL-1β (ab197742, Abcam, USA), IL-6 (ab222503, Abcam, USA) and TNF-α (ab208348, Abcam, USA).

### Immunofluorescence Staining

The brain slides were probed using immunofluorescence (IF) staining technique (Li et al. 2019, 2020). First, the mice were deep anesthetized and had transcardial perfusion with PBS (0.01 M, pH 7.40, 4 ℃) and then paraformaldehyde (PFA) solution (4%, pH 7.40, 4 ℃). The brain was immerged in the same PFA solution for 48 h at 4 ℃. After fixation, the brain was thoroughly dehydrated in saturated sucrose solution for 72 h. Second, the brain was embedded with freezing reagent and frozen at -80 ℃ for further sectioning. Third, the hardened brain was sectioned into brain slices with the thickness of 8 µm which were mounted to the glass slides. The brain slides were washed and then incubated with Triton X-100 and bovine serum (5%) for 1 h before IF staining. The primary antibodies and the following secondary antibodies were added to the brain slices to co-incubate as described. After washing with PBS, the slides were dried and given one drop of DAPI, and then were carefully sealed with glass. Finally, the slides were taken photos under a fluorescence microscope by an independent researcher. For analyzing the expression of GBNMB, five slides from the same brain specimen shall each have four different fields (200 ×) of vision examined, focusing mainly on the nearby areas of the perforating spot on the left cortex.

### Study Design

The grouping and use of mice were listed in the supplemental materials (Table S1).

Experiment 1: the trend of variation of GPNMB (n = 40).

The mice were randomly grouped into sham (n = 6), SAH 3 h (n = 6), SAH 6 h (n = 6), SAH = 12 h (n = 6), SAH 24 h (n = 6) and SAH 72 h (n = 6), 36 in total. WB was used to detect and analyze the protein expression of GPNMB before and after SAH. Four mice had IF staining to determine the expression and distribution situs of GPNMB in brain tissue.

Experiment 2: the effect of GPNMB (n = 144).

Five groups were established including sham (n = 6), SAH + vehicle (n = 6), SAH + GPNMB (1 µg/10 µL) (n = 6), SAH + GPNMB (3.3 µg/10 µL) (n = 6), and SAH + GPNMB (10 µg/10 µL) (n = 6) to study the effect of GPNMB on SAH and explore the dose–effect. SAH grading, mNSS, Garcia test, BWC and BBB integrity were assessed at 24 h and 72 h after SAH. Twenty-four mice were randomly assigned into three groups: sham (n = 8), SAH + vechicle (n = 8) and SAH + GPNMB (properly minimal dose, n = 8). The Rotarod test and Morris water maze test began to be performed before SAH (baseline) and respectively at 1 wk and 3 wk after SAH to evaluate the mid- and long-term neurofunction.

Experiment 3: the signaling pathway of the effect of GPNMB (n = 24).

Twenty-four mice were randomly grouped to sham (n = 6), SAH + vehicle (n = 6), SAH + GPNMB (n = 6) and SAH + Dorsomorphin (n = 6). WB was used to detect and analyze the protein expression of p-AMPK/AMPK, p-NFκB, IL-1β, IL-6 and TNF-α, attempting to reveal the signaling pathway by which the GPNMB affects the inflammatory response. Enzyme linked immunosorbent assay (ELISA) method was adopted to repeat the detection of aforementioned inflammatory related proteins to further confirm the signaling pathway.

### Statistical Analyses

All the statistical analyses were conducted using GraphPad Prism 9.3.3 (GraphPad Software, Inc., La Jolla, USA). The test for data distribution was conducted by Kolmogorov–Smirnov and Shapiro–Wilk test. Those conforming to normal distribution were processed with one-way or two-way analysis of variances (ANOVA), the statistical results were presented as mean ± standard deviation (SD). Data with abnormal distribution were analyzed using Wilcoxon test; the results were presented as median ± standard deviation (SD). All the results were considered significant if the *p* value less than 0.05 (*p* < 0.05).

## Results

### Mortality Rate

The mortality rate in sham group was 0 (0/46) whereas the overall mortality rate in SAH group was 6.9% (12/174). However, the mortality amongst the SAH groups was no significant.

### The Expression of GPNMB in Brain Tissue

**Fig. 1:**
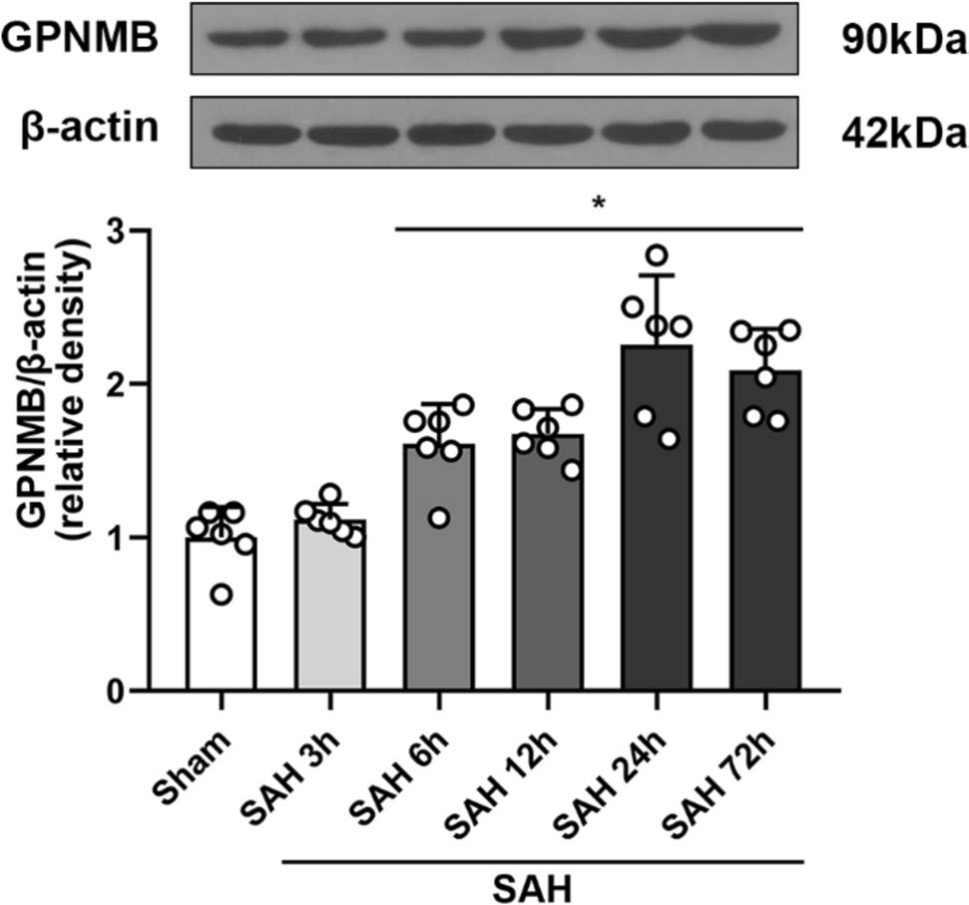
Protein expression of GPNMB. It showed that the expression of GPNMB rose significantly and the differences could be detected 6 h after SAH induction, ANOVA, *p < 0.05 vs. sham group, n = 6 / group

**Fig. 2:**
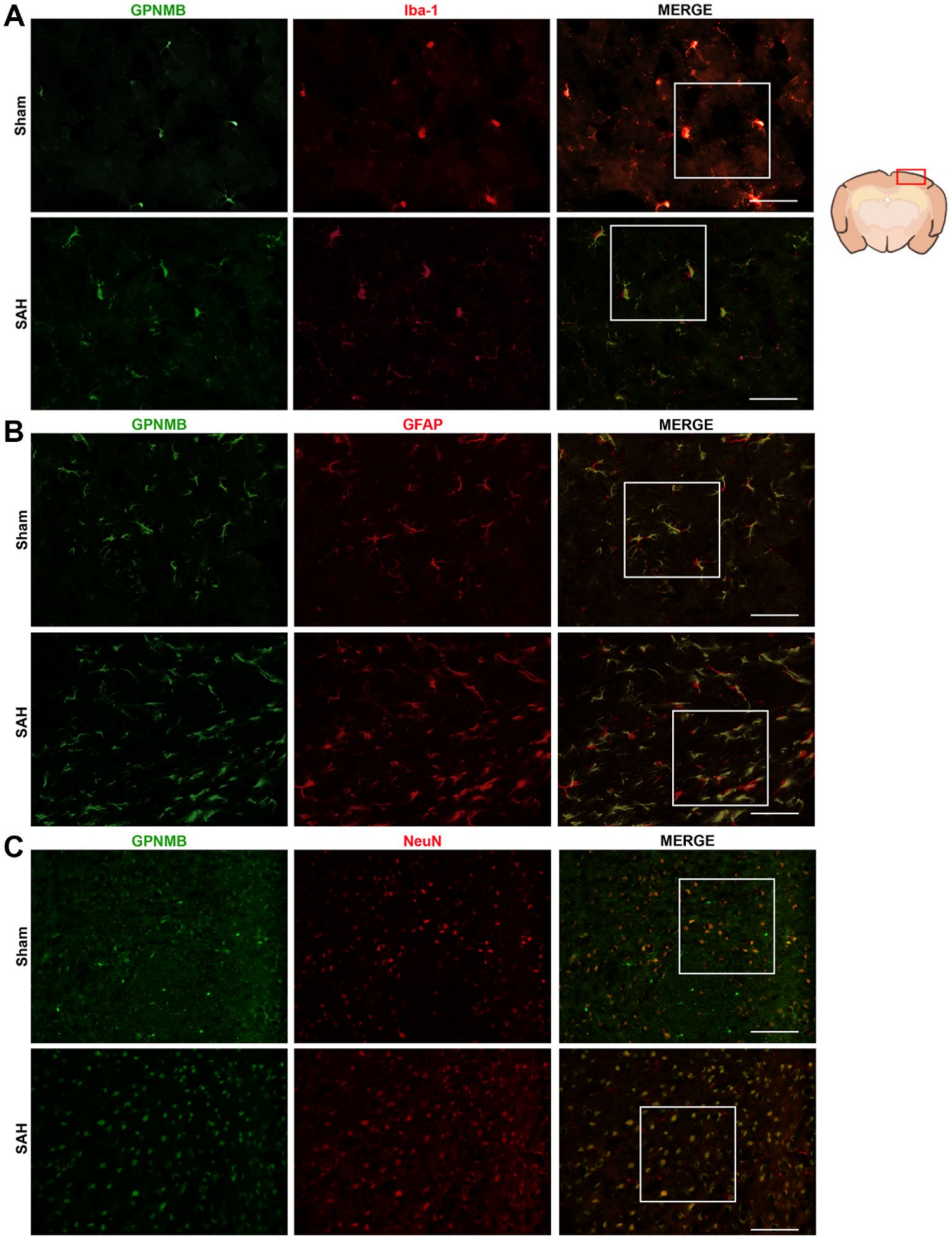
Immunofluorescence (IF) staining for GPNMB. GPNMB (green) costaining with **A** microglia (red), **B** astrocytes (red) and **C** neurons (red), from it we could see that GPNMB was expressed abundantly in microglia, astrocytes and neurons; all fields of vision were taken on the left cortical and subcortical areas, n = 2 / group

Results of WB showed that GPNMB increased significantly at 6 h after SAH induction and reached the apex at 24 h (*p* < 0.05, Fig. 1). It is suggested by the IF staining that GPNMB expressed extensively in microglia, astrocytes and neurons (Fig. 2a-c).

### SAH Grading and Short-term Neurofunction

**Fig. 3:**
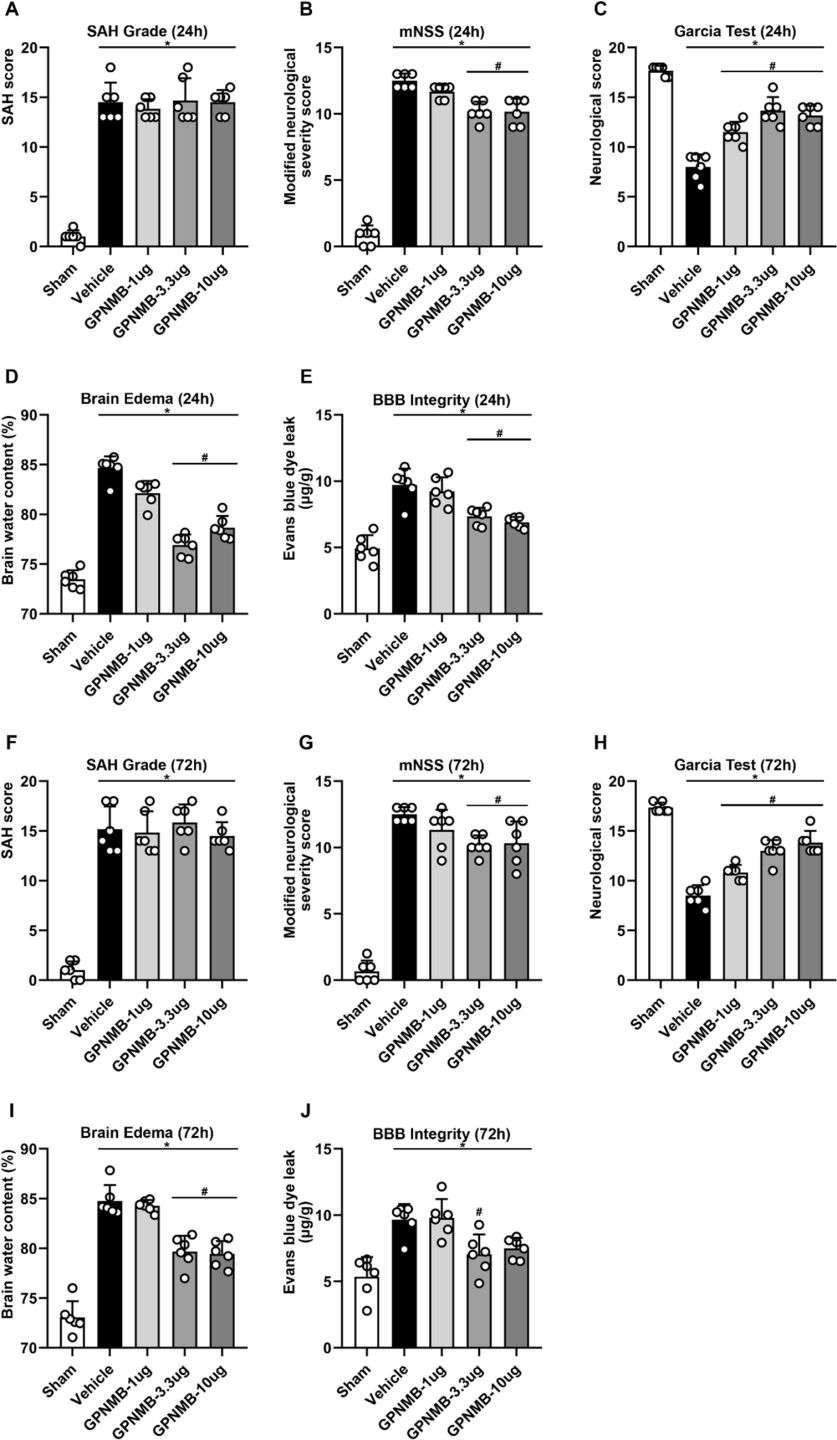
Gross assessment at 24 h and 72 h. (**A**) SAH grade indicated that all mice suffered from severe (≥ 13) SAH, and no difference was found among the SAH groups even administered with GPNMB. Anyhow, (**B**) mNSS and (**C**) Garcia test showed that neuronal function was compromised significantly after SAH induction and could be rescued by using GPNMB. Similarly, (**D**) BWC and (**E**) BBB integrity analyses showed that brain edema and BBB damage were significant after SAH, and administration of GPNMB had a protective effect especially at medium and high dose; (**F**) SAH grade results suggested the severe SAH, but no significances were noted among the vehicle and GPNMB groups. Results from (**G**) mNSS and (**H**) Garcia test showed that neurofunction was significantly impaired after SAH, and it was improved with GPNMB use. Additionally, (**I**) BWC and (**J**) BBB integrity had a significant defect after SAH, and were to some extent protected by using GPNMB, ANOVA, *p < 0.05 vs. sham group, #*p* < 0.05 vs. vehicle group, n = 6 / group

No blood in brain tissue was found in sham group (SAH grade = 0), while the degree of SAH in SAH groups was all severe (SAH grade ≥ 13) (*p* < 0.05, Fig. 3a). However, there was no significance noted among different SAH groups even in the GPNMB treatment groups. In terms of mNSS at 24 h, the score was significantly increased after SAH while had a reduction when GPNMB was administered (*p* < 0.05, Fig. 3b), especially on the medium and the highest dose. Likewise in Garcia test, the neuroscore was significantly decreased when SAH was induced, yet had an increase with GPNMB administration (*p* < 0.05, Fig. 3c). BWC significantly surged and BBB extravasation increased when SAH occurred, and were reversed by using GPNMB especially at concentration of 3.3 µg/10 µL and 10 µg/10 µL (*p* < 0.05, Fig. 3d-e).

The tests were repeated at 72 h after SAH, and the results were consistent with that at 24 h (*p* < 0.05, Fig. 3f-j). Though the results were not completely consistent with each comparison at 72 h and between 72 and 24 h, generally, it turns out that GPNMB administration could significantly improve the neurological outcomes at short-term, and the concentration of 3.3 µg/10 µL could be adopted as the optimal dosage for the following experiments.

### Mid- and Long-Term Neurofunction

**Fig. 4:**
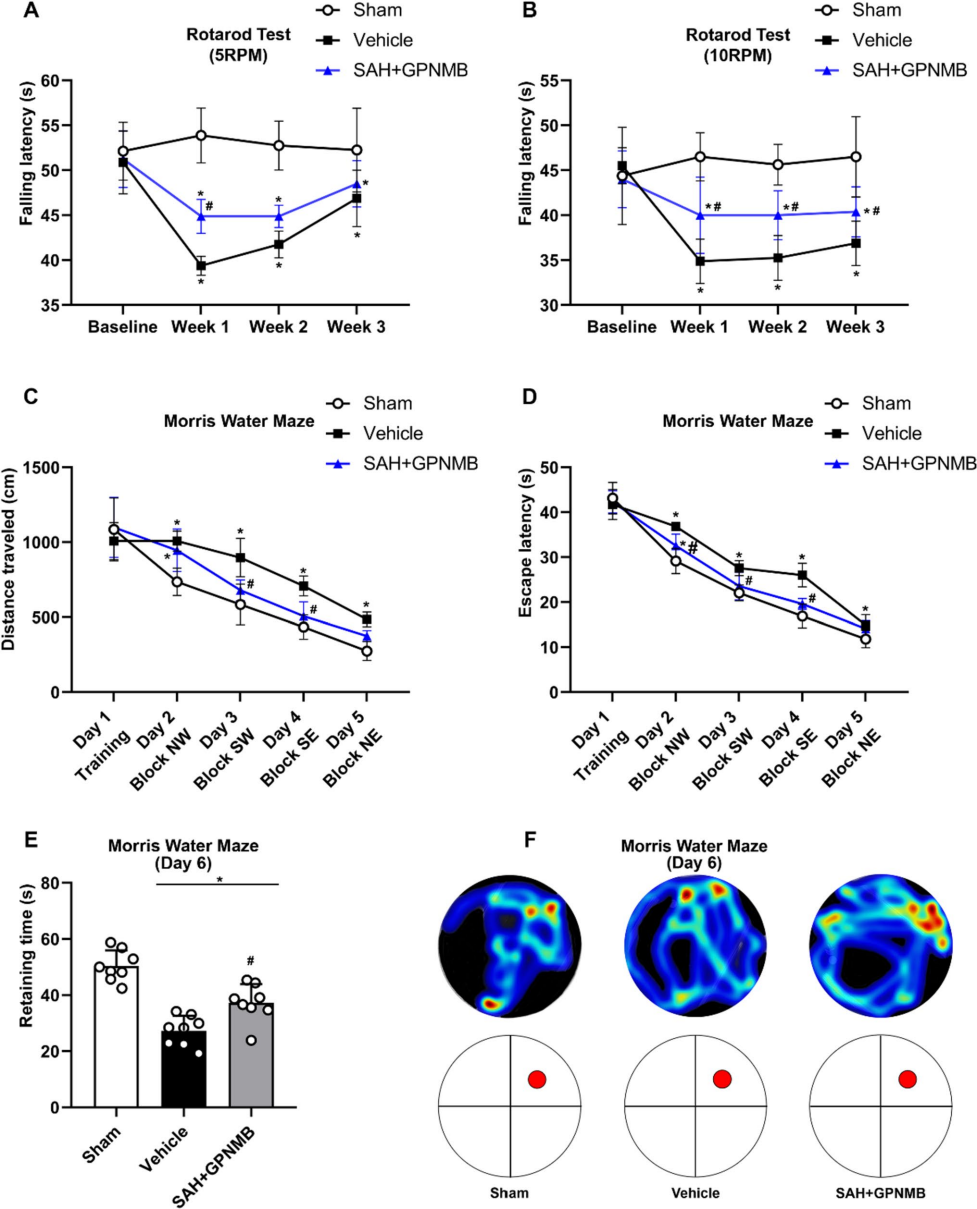
Mid- and long-term neurofunction tests. Rotarod test at (**A**) 5 RPM and (**B**) 10 RPM suggested that stable duration was significantly decreased after SAH and increased with the administration of GPNMB. Morris water maze test showed that (**C**) distance traveled and (**D**) escape latency was significantly dropped after SAH while had an increase with GPNMB usage. Also, (**E**) retaining time in the target quadrant was improved significantly in GPNMB group, and this was consistent with the (**F**) heatmap demonstration, ANOVA, *p < 0.05 vs. sham group, #*p* < 0.05 vs. vehicle group, n = 6 / group

Rotarod study showed that all the mice had no differences on capability of sensorimotor coordination and motor learning at the baseline; the stability dropped significantly after SAH, and the group treated with GPNMB had a significant increase in mid-term neurofunction (p < 0.05, Fig. 4a-b). Test results of Morris water maze indicated that all the groups of mice manifested no significance of cognition on the baseline. After SAH, the mice had a significant reduction on long-term neurofunction: the distance traveled, the escape latency, and retaining time in target block were all decreased significantly; the group administered with GPNMG had a significant elevation in these results (*p* < 0.05, Fig. 4c-e), and was evident in the improvement in the cognitive function (Fig. 4c-f).

### Mechanism of the Effect of GPNMB

**Fig. 5:**
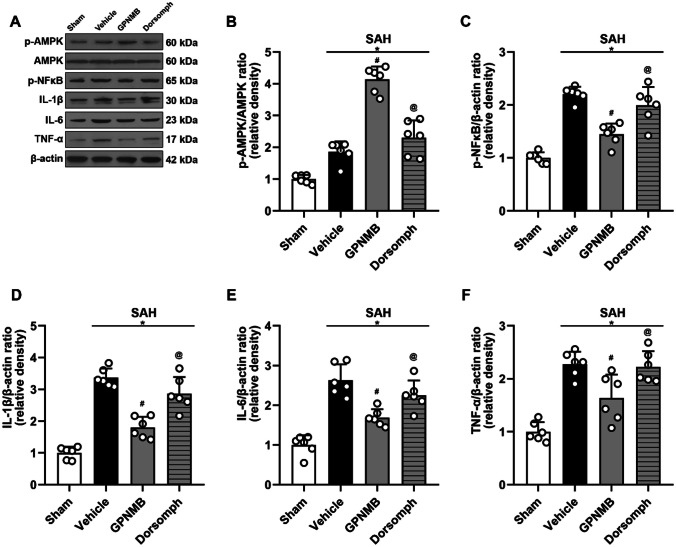
Western blot (WB) analyses of the proteins. **A** Representative bands and the results of **B** p-AMPK/AMPK ratio, **C** NFκB/β-actin ratio, **D** IL-1β, **E** IL-6 and **F** TNF-α suggested that p-AMPK/AMPK ratio, NFκB and the inflammatory cytokines all increased after SAH, and the administration of GPNMB could significantly increase the p-AMPK/AMPK ratio whereas decrease NFκB and the expression of the inflammatory cytokines. Furthermore, these effects could be reversed by AMPK inhibitor (Dorsomorphine), ANOVA, *p < 0.05 vs. sham group, #*p* < 0.05 vs. vehicle group, @*p* < 0.05 vs. GPNMB group, n = 6 / group

WB results suggested that p-AMPK/AMPK, p-NFκB, IL-1β, IL-6 and TNF-α increased significantly after SAH (Fig. 5a); it is significant that the pro-inflammatory proteins in mice treated with GPNMB decreased. Nonetheless, the group simultaneously administered with Dorsomophin had a reversed effect of GPNMB significantly, implying that GPNMB exerted the anti-inflammatory effect though the APMK/NFκB dependent signaling pathway (p < 0.05, Fig. 5b-f).

**Fig. 6:**
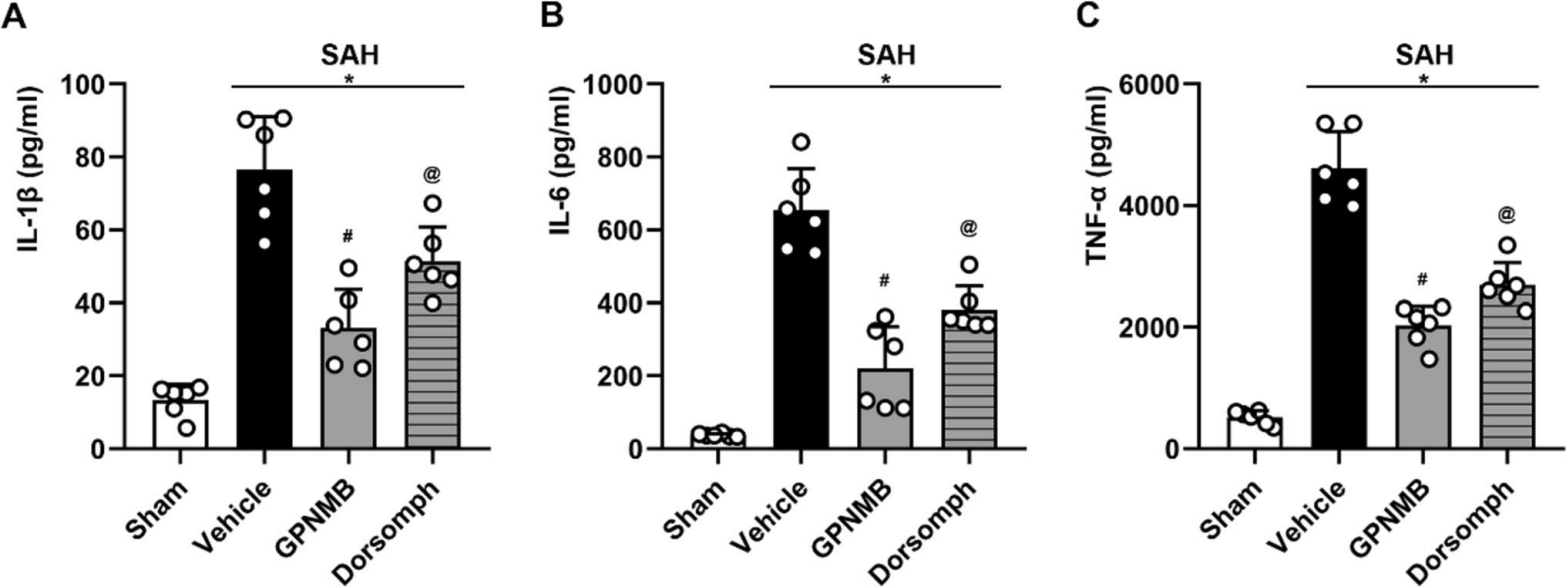
Enzyme linked immunosorbent assay (ELISA) results. ELISA was utilized to additionally confirm the expression of **A** IL-1β, **B** IL-6 and **C** TNF-α, and the results were consistent with that from WB that the expression of the inflammatory cytokines surged after SAH and could be decreased with GPNMB, ANOVA, *p < 0.05 vs. sham group, #*p* < 0.05 vs. vehicle group, @*p* < 0.05 vs. GPNMB group, n = 6 / group

We utilized the ELIZA method to further confirm the above results and yield the consistent conclusion that the protein expression of IL-1β, IL-6 and TNF-α significantly rose after SAH, and the elevation could be effectively inhibited by using GPNMG treatment. In the meantime, the selective AMPK inhibitor, Dorsomophin could reverse such an effect of GPNMB (*p* < 0.05, Fig. 6a-c).

## Discussion

We have found that GPNMB is involved in the course of SAH; its expression is increased after SAH and abundant in microglia, astrocytes and neurons. Exogenous administration of GPNMB can exert a neuroprotective effect, specifically, increase neurofunctional score, decrease BWC and prevent BBB from further damaging. In addition, using GPNMB in time after SAH can also offer mid- and long- term neurofunctional protection. The molecular mechanisms underlying the beneficial effects of GPNMB appear to involve the modulation of AMPK and NFκB signaling pathways. The activation of AMPK is generally associated with protective cellular responses, including anti-inflammatory actions (Kolb et al. 2021). On the other hand, NFκB is known to play a critical role in the initiation of the inflammatory response (Li et al. 2001). The fact that GPNMB treatment increased the activation of AMPK and decreased the activation of NFκB is consistent with its observed neuroprotective effects.

However, it's important to note that when GPNMB treatment was combined with an AMPK inhibitor, we observed a reversal of these molecular changes, along with an increase in inflammatory cytokines. This strongly suggests again that the anti-inflammatory effects of GPNMB are mediated, at least in part, by modulation of AMPK and NFκB signaling. Future studies will be needed to fully dissect the molecular mechanisms involved.

Conventional theory recognizes the brain injury brought by SAH is basically due to cerebral vasospasm and the consequent brain ischemia. However, research and clinical practice based on this are all in vain. To explain the phenomena and explore the mechanisms, EBI which covers not only cerebral vasospasm but also low cerebral perfusion pressure, increased intracranial pressure, oxidative over-stress, immune injury, apoptosis, ferroptosis, neuroinflammation and white matter injury has been proposed in recent years (Fujii et al. 2013; Sehba et al. 2012). Alleviation of EBI is the key to the treatment of SAH. Study results in our work suggested that rGPNMB had an anti-inflammatory effect possibly via the AMPK/NFκB signaling pathway to decrease the downstream inflammatory cytokines, thus exhibited a neuroprotective approach towards the experimental mice suffered from SAH. GPNMB ameliorated the EBI and improved the neurofunction at the acute stage (≤ 72 h), furthermore, it showed the potential to improve the mid- and long-term neurofunction. That is consistent with the core idea of EBI theory, which proposes that the improvement of EBI, for example ameliorating the inflammatory response in the pathological processes, could not only amend the acute injury but also have a protective effect for mid-and long-term neurofunction. If these findings can be replicated and extended in further studies, GPNMB might become a novel target for therapeutic intervention.

Although the effect and relevant mechanism of GPNMB have been reported in this study pioneeringly. There are some limitations of our study. Firstly, we revealed that GPNMB may have the effect via the AMPK/NFκB signaling, but how the exogenous rGPNMB works on the surface receptors to activate intra-cellular effectors remains unexplored. Secondly, it is suggested by numerous studies that GPNMB plays the anti-inflammatory effect through multiple mechanisms, AMPK/NFκB signaling may be one of them. Thirdly, GPNMB has a variety of physiological and pathological effectiveness on diseases in central nervous system including hemorrhagic stroke, anti-neuroinflammation is one of the perspectives. Lastly, the study was conducted in mice, and although mice models are often used in preliminary studies, results may not entirely extrapolate to humans. Therefore, more studies on GPNMB in other models, including larger mammals or human cells demand to be executed in the future.

In conclusion, our study has revealed a potentially significant role for GPNMB in the neuroinflammatory response following SAH. Further investigation into this glycoprotein's therapeutic potential could pave the way for new treatment options for patients suffering from SAH and potentially other neuroinflammatory conditions.

### Supplementary Information

Below is the link to the electronic supplementary material.

Supplementary file1 (DOCX 17 KB)

## Data Availability

The data and the analyzing sheets of this study are available from the corresponding authors, and we are willing to share them under reasonable circumstances.
