# Supplementary material for: GPNMB Ameliorates Neuroinflammation Via the Modulation of AMPK/NFκB Signaling Pathway After SAH in Mice
Source: J Neuroimmune Pharmacol. 2023 Nov 3;18(4):628–39. doi: 10.1007/s11481-023-10087-6 (PMC10769934; doi:10.1007/s11481-023-10087-6)
Supplement: Supplementary file 1 — Supplementary file1 (DOCX 17 KB) [file 11481_2023_10087_MOESM1_ESM.docx]

| **Groups** | **Mortality** | **Excluded** |
| --- | --- | --- |
| **Experiment 1: the trend of variation of GPNMB** |  |  |
| Sham (n=8) | 0 (0/8) | 0 |
| SAH (3h, 6h, 12h, 24h, 72h) | 5.9% (2/34) | 0 |
| **Experiment 2.1: short-term outcome study (24h and 72h)** |  |  |
| Sham (n=24) | 0 (0/24) | 0 |
| SAH+Vehicle (n=26) | 7.7% (2/26) | 2 |
| GPNMB 1ug/10ul (n=27) | 11.1% (3/27) | 0 |
| *GPNMB 3.3ug/10ul (n=25) | 4.0% (1/25) | 1 |
| GPNMB 10ug/10ul (n=26) | 7.7% (2/26) | 0 |
| **Experiment 2.2: long-term outcome study** |  |  |
| Sham (n=8) | 0 (0/8) | 0 |
| SAH+Vehicle (n=8) | 0 (0/8) | 0 |
| SAH+GPNMB (n=8) | 0 (0/8) | 0 |
| **Experiment 3: signaling pathway** |  |  |
| Sham (n=6) | 0 (0/6) | 0 |
| SAH+Vehicle (n=7) | 14.3% (1/7) | 1 |
| SAH+GPNMB (n=7) | 14.3% (1/7) | 0 |
| SAH+GPNMB+Dorsomph (n=6) | 0 (0/6) | 0 |
| **TOTAL** |  |  |
| Sham | 0 (0/46) | 0 |
| SAH | 6.9% (12/174) | 4 |

**Table S1 Mice Grouping and Used in the Studies**

*GPNMB 3.3ug/10ul was the optimal dose and adopted for the following studies.

A total of 220 mice were used: 46 in sham group, 174 in SAH group. Nonetheless, 4 mice were excluded due to fail to establish the SAH model.
